# The development of search filters for adverse effects of medical devices in medline and embase

**DOI:** 10.1111/hir.12260

**Published:** 2019-06-11

**Authors:** Su Golder, Kelly Farrah, Monika Mierzwinski‐Urban, Kath Wright, Yoon Kong Loke

**Affiliations:** ^1^ Department of Health Sciences University of York York UK; ^2^ CADTH Ottawa, Ontario Canada; ^3^ CRD University of York York UK; ^4^ Norwich Medical School University of East Anglia Norwich UK

**Keywords:** embase, information retrieval, literature searching, medical subject headings (MeSH), medline, meta‐analysis, methodological filters, review, search strategies

## Abstract

**Background:**

Objectively derived search filters for adverse *drug* effects and complications in surgery have been developed but not for medical device adverse effects.

**Objective:**

To develop and validate search filters to retrieve evidence on medical device adverse effects from ovid medline and embase.

**Methods:**

We identified systematic reviews from Epistemonikos and the Health Technology Assessment (hta) database. Included studies within these reviews that reported on medical device adverse effects were randomly divided into three test sets and one validation set of records. Using word frequency analysis from one test set, we constructed a sensitivity maximising search strategy. This strategy was refined using two other test sets, then validated.

**Results:**

From 186 systematic reviews which met our inclusion criteria, 1984 unique included studies were available from medline and 1986 from embase. Generic adverse effects searches in medline and embase achieved 84% and 83% sensitivity. Recall was improved to over 90%, however, when specific adverse effects terms were added.

**Conclusion:**

We have derived and validated novel search filters that retrieve over 80% of records with medical device adverse effects data in medline and embase. The addition of specific adverse effects terms is required to achieve higher levels of sensitivity.

**Key Messages**

- Searches with generic adverse effects terms as suggested in this paper achieve over 80% relative recall in either medline or embase.
- The addition of specific named adverse effects search terms in either medline or embase is likely to improve relative recall to over 90%.
- Searching with adverse effects terms is unlikely to achieve 100% recall as some records do not indicate that the full paper contains adverse effects data.
- The relative recall achieved from searching with adverse effects terms for medical devices is slightly lower to that for drug interventions and surgical procedures.

## Introduction

Systematic reviews usually employ highly sensitive search strategies that aim to identify as many relevant papers as possible. However, retrieving a complete data set of studies on adverse effects is challenging due to inconsistent terminology and poor reporting (Golder, McIntosh, Duffy & Glanville, 2006). Medical devices are equipment, instruments, software or related articles intended for use in health care; they include stents, the contraceptive coil, breast implants and hip replacements. Retrieving studies on non‐drug interventions such as medical devices is particularly challenging because the primary studies are less likely to have incorporated adverse effects data and may be smaller than studies of drug interventions, making event data more sparse and their retrieval more difficult (Golder, Wright & Loke, 2017). For medical devices, in particular, adverse effects are more likely to be overlooked or not considered important. Even when they are considered they are likely to be secondary or tertiary outcomes. This may be due to the regulatory requirements for research evidence on the safety of new devices being universally less stringent than those for medicines (Golder & Loke, 2012a,b,c). The reporting and terminology surrounding adverse effects in medical devices have also been notoriously inconsistent, and this is reflected in the indexing of database records. In addition, as with other interventions, not all adverse effects may be known at the time of searching and it is common to include study designs beyond randomised controlled trials (RCTs) for identifying the adverse effects of medical devices. Whilst search filters for RCTs have been proven to perform well, searching for non‐RCT study designs is more problematic (Higgins & Green, 2011).

One way to help enable efficient searching for adverse effects could be through the development of search filters. Search filters are combinations of search terms which are designed to improve the efficiency and effectiveness of searching. Search filter development for adverse effects has tended to concentrate on identifying studies that report on adverse *drug* effects (Badgett, Chiquette, Anagnostelis & Mulrow, 1999; Golder & Loke, 2012a,b,c; Golder et al., 2006; Wieland & Dickersin, 2005). However, a different approach is required for the adverse effects of medical devices (Farrah, Mierzwinski‐Urban & Cimon, 2016; Golder, Wright & Rodgers, 2014; Golder et al., 2017). The different search strategies required for medical devices as opposed to drug adverse effects has been demonstrated by the poor retrieval obtained when our adverse drug effect search filter (which obtains between 89% and 97% of the relevant drug literature) (Golder & Loke, 2012b,c), identified only 54% of the literature on the adverse effects of medical devices (Farrah et al., 2016).

Search filters may be useful not only for librarians and information professionals but also for clinicians, researchers, guideline producers and policymakers. A relatively efficient method of retrieving useful information would benefit all searchers not just expert searchers. Information is required to enable decision‐making in clinical practice to generate appropriate advice on the benefit:harm of medical devices.

The creation of a medical device adverse effect search filter would be particularly timely given the current developments in embase. Elsevier (who produce embase) have been improving the indexing for adverse effects of medical devices in a number of ways. In 2014, they introduced the subheading ‘adverse device effect’, and by April 2018, this had been used in the indexing of 30 000 records. In addition, Elsevier have added further EMTREE indexing terms for medical devices – for example, endoscopes, catheters and prostheses and now have over 3000 specific terms.

We aimed to create highly sensitive validated search filters for ovid medline and embase to identify studies on medical device adverse effects.

## Methods

### Systematic review identification

Systematic reviews of adverse effects were identified by searching Epistemonikos (https://www.epistemonikos.org/) and the Health Technology Assessment (hta) database via ovid. Epistemonikos was chosen as it is currently the largest source of systematic reviews still being updated. Similarly, the hta database is the largest source of technology assessments from around the world.

Due to the large volume of systematic reviews published in the years 2015–2017, we were unable to simply sift the records available in Epistemonikos. We therefore conducted a series of searches for named ‘medical devices’ in combination with terms relating to ‘safety’. Searches were conducted on the 20 and 21 June 2017 and Publication Type: Systematic Reviews. A limit was placed of ‘Publication Date: 2015 to 2017’ in order to retrieve a recent cohort of systematic reviews. Additionally, the size of the sample needed to be restricted because of resource constraints. The safety terms were derived from previous research (Golder et al., 2006) and the medical device terms from a list of device terms provided by Elsevier (Box A1). The hta database was searched with the search strategy (‘2015’ or ‘2016’ or ‘2017’).di on the 23 June 2017.

Box A1 **Search strategy in Epistemonikos**

safe*OR complication*OR adverse*OR side effect*OR harm*OR risk*OR tolerate*OR sequelae.

AND

revascularization OR defibrillator*OR aortic aneurysm endovascular graft OR surgical mesh OR levonorgestrel releasing intrauterine system OR balloon OR plate OR mask OR device*OR wire*OR ventilator*OR equipment OR coil OR tube OR stocking*OR stapler*OR stent*OR plug*OR catheter*OR stoma OR suture*OR pacemaker*OR implant*OR electrode*OR endoprosthesis*OR laser*OR sling*OR screw*OR scaffold*OR clip*OR hearing aid*OR electronic cigarette OR glue OR gastric band OR pump*OR fixator*OR Spacer*OR microcatheter*OR orthosis OR tape OR trocar OR ring OR filter*OR videolaryngoscope OR valve*OR arthrometer, needle*OR bandage*OR dressing*OR nail*OR pin OR bone plating system OR pins OR brace OR collar*OR colonoscope OR condom OR battery OR generator OR sleeve*OR monitor OR monitors OR neurostimulator*OR keratoprosthesis OR morcellator OR instrument*OR cannula OR laryngoscope*OR navigation system*OR regulating system*OR cage*OR crown*OR patch*OR shunt*OR snare*OR clamp OR occluder*OR drain*OR adhesion OR plug*OR bypass OR artificial OR defibrillator*OR enema OR bath OR bioprosthesis OR distractor*OR staple OR bronchoscope OR camera OR lavage system*OR bag OR computer system*OR lens OR abutment OR endoscope OR dissector*OR inhaler*OR duodenoscope OR embolectomy system OR endobronchial blocker*OR esophageal bougie OR esophageal dilator*OR apparatus OR fluoroscopy system OR glove OR forcepts OR head holder OR sphincter*OR morcellator*OR stimulator*OR infusion system lithotripter*OR manikin OR mobile phone OR mouth gag OR shell*OR operating room OR operating table OR osteosynthesis material OR protective clothing OR scanner*OR humidifier*OR robotic*OR scalpel*OR sigmoidoscope OR splint OR drill*OR microscope*OR pulsation system OR adhesive*OR expander*OR flowmeter*OR cap OR pessary OR pessaries OR wheelchair* (where * is truncation symbol).

A systematic review was considered eligible for inclusion if:

- Adverse effect(s) for a medical device were the primary or secondary outcome. The device was required to be the main focus of the review. If the review focused more heavily on the surgical procedure needed to implant the device or the drug component of the device (such as anticoagulation after stenting) or was focused on prevention of adverse effects, it was excluded on this basis. The World Health Organisation (WHO) definition of a medical device was used: ‘“Medical device” means any instrument, apparatus, implement, machine, appliance, implant, reagent for *in vitro* use, software, material or other similar or related article, intended by the manufacturer to be used….for specific medical purpose(s)’ http://www.who.int/medical_devices/full_deffinition/en/
- The search strategy was reported in the published paper, and no adverse effects search terms (either generic, such as ‘adverse effects’ or ‘side‐effects’ or named, such as ‘fatigue’ or ‘insomnia’) had been used. Typically, such reviews rely on terms for the population or condition and intervention only. This enabled us to construct an unbiased cohort which did not include articles that had been retrieved because they already contained adverse effects terms.
- The search included either handsearching or reference checking in addition to database searches. This was in an attempt to compensate for potential deficiencies in the search strategies.
- At least one included study was related to safety. This was because some reviews were unsuccessful in retrieving any relevant studies.

We excluded reviews that (a) were in a non‐English language – which we were unable to obtain a translation for and (b) where the full text was unavailable.

Two researchers independently screened titles and abstracts using Distiller and selected systematic reviews for potential inclusion. Any discrepancies between the researchers were resolved by discussion and consensus or by a third reviewer. The full text of potentially relevant systematic reviews was also independently screened, with discrepancies resolved by discussion and consensus.

### Included primary studies

The full text of the included articles within these systematic reviews was checked to confirm the presence of adverse effects data. The use of included papers from systematic reviews has been shown to be an effective alternative to handsearching to identify a reference standard set of records for developing and evaluating search strategies (Sampson et al., 2006).

The first stage of the analysis was to check whether each paper was contained in medline or embase. We used several search iterations as necessary of the author names or words from the paper to identify each record. The records available on medline and embase were then divided into three test sets and one validation set of records using random numbers generated by RANDOM.ORG.

Individual word and multiple‐word frequency analysis on the first test set of records was undertaken using WriteWords to identify commonly occurring terms related to adverse effects. WriteWords is freely available on the Internet and allows frequency counting of the usage of words or phrases (http://www.writewords.org.uk/phrase_count.asp). We calculated relative recall as a measure of the percentage of known records retrieved using the filter because it provides an estimate of sensitivity (Sampson et al., 2006). The relative recall of the relevant search terms was calculated using the following formula:

#### Relative recall calculation

$$\begin{array}{cc} & \frac{\text{No of relevant records retrieved}}{\text{No of relevant records available}}\\ & \times 100=\text{Relative recall as a percentage}\;(\%). \end{array}$$

A draft filter was created with the first test set. We started with the search term that had the highest recall and then tested all other potentially relevant terms to ascertain the incremental increase in recall when added to the first search term. This process continued until no more new records were being identified by additional search terms.

The filter created with the first test set was next applied to the second test set, then after any additional modifications, such as additional search terms, the filter was applied to the third test set. After any further modifications from applying the filter to the third test set, the retrieval performance of the search filter was tested in the validation set.

We also examined those records not retrieved by our generic search term filters to ascertain whether specific adverse effects search terms (such as ‘infection’ or ‘mortality’) would have been successful in the retrieval of additional records. We noted any database records with no indication that the full text contained information on adverse effects.

In order to give a relative or rank estimate of the precision of the search terms, we also identified the total number of records retrieved from medline or embase at the time of conducting the present research using each search term. We then calculated an approximation of the *relative* precision of the term in comparison with the other terms we identified.

This whole process was first undertaken in medline and then repeated in embase.

## Results

**Figure 1:**
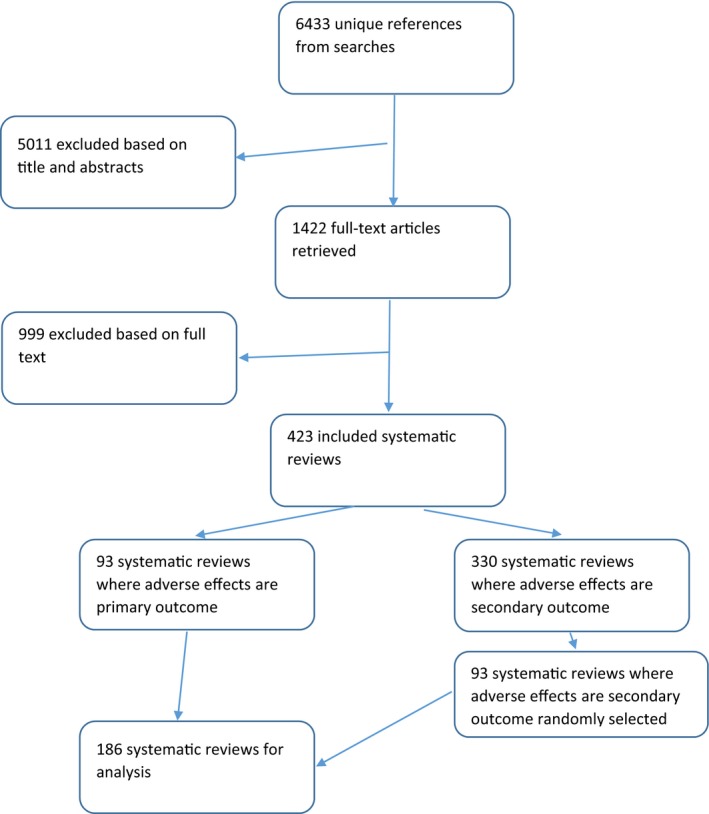
Flow diagram [Colour figure can be viewed at http://wileyonlinelibrary.com]

From 6433 records screened, 1422 full‐text reports were retrieved of which 423 met our inclusion criteria. Of these 423 reviews, 93 were systematic reviews where the primary outcome was an adverse effect(s) of a medical device and 330 systematic reviews had adverse effects as secondary outcomes. Due to constraints on time and resources, we limited the analysis to the 93 reviews with adverse effects as a primary outcome and a random selection of 93 of the 330 reviews with adverse effects as a secondary outcome – giving a total of 186 reviews (Figure 1). These 186 reviews included 2130 studies (2278 studies before deduplication) and of these included studies – 1984 unique records were available on medline and 1986 on embase.

### medline

The gold standard set of 1984 records in medline were randomly allocated into three test sets of 496 records each and one validation set of 496 records.

#### First test set for the development of the medline search filter

Of the search terms identified in the first test set – ‘complicat*’ in the title and abstract had the highest recall and was searched first. This was followed by the floating subheading ‘adverse effects (ae)’ which gave the highest incremental increase in recall when added to ‘complicat*’ in the title and abstract (Table A1 and Box 1).

Box 1 **medline search strategy from first, second and third test set of records**

**Table:** 

Test Set 1	Test Set 2	Test Set 3
complicat*.ti,ab. (196) ae.fs. (290) safe*.ti,ab. (333) exp postoperative complications/(368) failure*.ti,ab. (392) adverse.ti,ab. (403) co.fs. (412) failed.ti,ab. (420) exp equipment failure/(426) removal.ti,ab.(431) equipment safety/(433) problem*.ti,ab. (435) side effect*.ti,ab.(436) harmful.ti,ab. (437) tolerated.ti,ab. (438) loosen*.ti,ab. (439) OR/1‐16	complicat*.ti,ab. ae.fs. safe*.ti,ab. exp postoperative complications/ failure*.ti,ab. adverse.ti,ab. co.fs. failed.ti,ab. exp equipment failure/ removal.ti,ab. equipment safety/ problem*.ti,ab side effect*.ti,ab. harmful.ti,ab. tolerated.ti,ab. loosen*.ti,ab. **Intraoperative Complications/** **migration.ti,ab.** breakag*.ti,ab. OR/1‐19	complicat*.ti,ab. ae.fs. safe*.ti,ab. exp postoperative complications/ failure*.ti,ab. adverse.ti,ab. co.fs. failed.ti,ab. exp equipment failure/ removal.ti,ab. equipment safety/ problem*.ti,ab. side effect*.ti,ab. Harmful.ti,ab. Tolerated.ti,ab. loosen*.ti,ab. Intraoperative Complications/ migration.ti,ab. breakag*.ti,ab. **discomfort.ti,ab.** **displacement.ti,ab.** detrimental adj2 effect*.ti,ab. **untoward effects.ti,ab.** OR/1‐23

The bold refers to new terms added to the search since the last iteration

The addition of further terms resulted in a search strategy (Box 1) which retrieved 89% (439/496) of records. Of the 57 records not retrieved – 25 contained terms for specific adverse effects (Table A2) whereas 32 records gave no indication that the full paper contained information on adverse effects. The specific adverse effects terms (such as sore throat and dysphagia) were not added to the search as they tended to only apply to specific medical devices. A search strategy which incorporates both generic and specific adverse effects terms could therefore potentially achieve 94% (464/496) recall.

The search terms which gave the highest precision in medline (Tables 1 and A2) were estimated to be ‘safety‐based medical device withdrawals/’ [MeSH], ‘medical device recalls/’ [MeSH] and ‘device removal/’ [MeSH]. The search terms with the best balance in precision and recall (Table 1 and Table A2) were estimated to be ‘exp equipment failure/’ [MeSH], ‘complications’ [Title/Abstract] and ‘complication*’ [Title/Abstract].

**Table 1:** Relative precision of search terms in medline and embase

medline terms with highest precision	medline terms with best balance in precision and recall	embase terms with highest precision	embase terms with best balance in precision and recall
safety‐based medical device withdrawals/[MeSH]	exp equipment failure/[MeSH]	adverse reaction to metal debris/[EMTREE]	adverse reaction to metal debris/[EMTREE]
medical device recalls/[MeSH]	complications [Title/Abstract]	device related events [Title/abstract]	exp medical device complication/[EMTREE]
device removal/[MeSH]	complication* [Title/Abstract]	device recall/[EMTREE term]	complication* [Title/Abstract]
failure*[Keyword heading]	device removal/[MeSH]	malfunction [Candidate term word]	medical device complication/[EMTREE]
Loosening [Title/Abstract]	complicat* [Title/Abstract]	complication [Candidate term word]	complications [Title/Abstract]
loosen* [Title/Abstract]	exp postoperative complication/[MeSH]	device removal [Title/Abstract]	device related events [Title/Abstract]
exp equipment failure/[MeSH]	failure* [Subject heading word]	medical device complication/[EMTREE]	exp complication/[EMTREE]
equipment failure/[MeSH]	medical device recalls/’ [MeSH]	device safety/[EMTREE]	device safety/[EMTREE]
equipment safety/[MeSH]	safe* [Title/Abstract]	equipment safety/[EMTREE]	exp device removal/[EMTREE]
malfunction* [Title/Abstract]	safety‐based medical device withdrawals/[MeSH]	device removal/[EMTREE]	device removal/[EMTREE]

#### Second test set for the development of the medline search filter

The search strategy from the first test set (Box 1) was tested on the second test set of records and retrieved 87% (432/496). On inspection of the records that had not been retrieved, we found three additional generic adverse effects terms – ‘intraoperative complications/’ [MeSH], ‘migration’ in the abstract, and ‘breakag*’ in the abstract. These additional terms were added to the search strategy, and 88% (438/496) of records were retrieved.

Of the 58 records that had not been retrieved by this search strategy, 28 contained specific adverse effects terms (Table A2). A search strategy which incorporates both generic and specific adverse effects terms could therefore potentially achieve 94% (466/496) recall in the second test set of records.

#### Third test set for the development of the medline search filter

The search strategy from the second test set (Box 1) was tested on the third test set of records and retrieved 89% (443/496) of records. On inspection of the records that had not been retrieved, we found additional generic adverse effects terms in the abstract, ‘detrimental adj2 effect*’, ‘discomfort’, ‘displacement’ and ‘untoward effects’. These terms were added to the search strategy, and 91% (450/496) records were retrieved.

Of the 46 records that had not been retrieved by this search strategy – 18 contained specific adverse effects terms (Table A2). A search strategy which incorporates both generic and specific adverse effects terms could therefore potentially achieve 94% (468/496) recall in the third test set of records.

#### Validation of the medline search filter

The revised search strategy (Box 1) performed less well on the validation set of records then in the test sets and retrieved 83% (414/496) of records. We conducted post hoc analysis to identify factors that may have affected the recall. There was one additional record that could have been retrieved if ‘post‐operative morbidity’ in the abstract was added to the search strategy.

Of the 82 records not retrieved, 40 contained terms related to specific adverse effects (Table A2). A search strategy which incorporates both generic and specific adverse effects terms could therefore potentially achieve 92% (454/496) recall in the validation set of records.

### embase

The gold standard set of 1986 records in embase were randomly divided into three test sets of 496 records each and a validation set of 498 records.

#### First test set of records for the development of the embase search filter

The floating subheading ‘complication (co)’ had the highest recall and was searched first. This was followed by ‘complicat*’ in the title and abstract which gave the highest incremental increase in recall when added to the floating subheading ‘complication (co)’(Table A3 and Box 2).

Box 2 **embase search strategy from first, second and third test set of records**

**Table:** 

Test Set 1	Test Set 2	Test Set 3
co.fs. (219) complicat*.ti,ab. (311) safe*.ti,ab. (354) failure*.ti,ab. (386) exp medical device complication/(395) adverse.ti,ab. (402) failed.ti,ab. (409) exp postoperative complication/(414) problem*.ti,ab. (419) side effect*.ti,ab.(422) discomfort.ti,ab. (425) loosen*.ti,ab. (428) removal*.ti,ab. (431) complications.kw. (433) migration.ti,ab. (435) ae.fs. (437) device related events.ti,ab. (438) adverse effects/(439) OR/1‐19	co.fs. complicat*.ti,ab. safe*.ti,ab. failure*.ti,ab. exp medical device complication/ adverse.ti,ab. failed.ti,ab. exp postoperative complication/ problem*.ti,ab. side effect*.ti,ab. discomfort.ti,ab. loosen*.ti,ab. removal*.ti,ab. complications.kw. migration.ti,ab. ae.fs. device related events.ti,ab. adverse effects/ **device safety/** **safety/** **peroperative complication/** **tolerated.ti,ab.** OR/1‐22 Line 19 could have been ‘equipment safety’ as a keyword instead or ‘device safety/’ to retrieve the same records. ‘Device safety/’ was selected due to its potentially higher precision.	co.fs. complicat*.ti,ab. safe*.ti,ab. failure*.ti,ab. exp medical device complication/ adverse.ti,ab. failed.ti,ab. exp postoperative complication/ problem*.ti,ab. side effect*.ti,ab. discomfort.ti,ab. loosen*.ti,ab. removal*.ti,ab. complications.kw. migration.ti,ab. ae.fs. device related events.ti,ab. adverse effects/ device safety/ safety/ peroperative complication/ tolerated.ti,ab. **failing.ti,ab.** OR/1‐23

The bold refers to new terms added to the search since the last iteration

The addition of further terms resulted in a search strategy (Box 2) which retrieved 89% (439/496) records. Of the 57 records not retrieved by the search strategy, 30 had terms related to specific adverse effects (Table A4) whereas 27 gave no indication that the full paper contained information on adverse effects. A search strategy which incorporates both generic and specific adverse effects terms could therefore potentially achieve 95% (469/496) recall.

The terms which gave the highest precision in embase (Table 2 and Table A3) were estimated to be ‘adverse reaction to metal debris/’ [EMTREE], ‘device related events’ [Title/abstract] and ‘device recall/’ [EMTREE] (Table 1). The search terms with the best balance in precision and recall (Table 1 and Table A3) were estimated to be ‘adverse reaction to metal debris/’ [EMTREE], ‘exp medical device complication/’ [EMTREE] and ‘complication*’ [Title/Abstract].

**Table 2:**
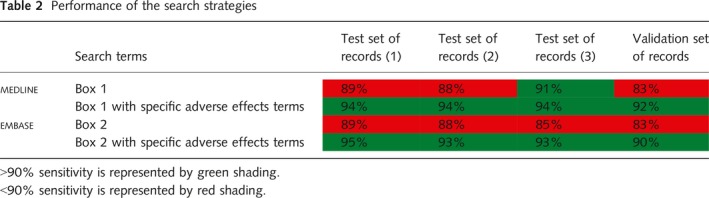


#### Second test set of records for the development of the embase search filter

The search strategy from the first test set (Box 2) was tested on the second test set of records and retrieved 87% (431/496). There were four additional records that could have been retrieved if ‘device safety/’ or ‘equipment safety’ as a keyword, ‘peroperative complication/’, ‘safety/’ and ‘tolerated’ in the abstract were added to the search strategy. After adding these terms to the search strategy – the revised strategy retrieved 88% (435/496) of the records in this second test set.

Of the 61 records not retrieved, 24 had terms related to specific adverse effects (Table A4). A search strategy which incorporates both generic and specific adverse effects terms could therefore potentially achieve 93% (459/496) recall in the second test set of records.

#### Third test set of records for the development of the embase search filter

The search strategy from the second test set (Box 2) was then tested on the third test set of records and retrieved 85% (423/496) of records. There were two additional records in this test set that could have been retrieved if ‘failing’ in the abstract was added to the search strategy. Hence, after adding the term ‘failing’ – the revised strategy retrieved 86% (425/496) of records in this third test set.

Of the 72 records not retrieved by the search strategy, 37 had terms related to specific adverse effects (Table A4). A search strategy which incorporates both generic and specific adverse effects terms could therefore potentially achieve 93% (462/496) recall in the third test set of records.

#### Validation of the embase search filter

The revised search strategy in Box 2 was then tested on the validation set of records and retrieved 410/498 (83%) of the records. We conducted post hoc analysis to identify factors that may have affected the recall. When we explored the records that had not been retrieved from the validation set, ‘postoperative complications/’ and ‘adverse drug reaction/’ and ‘high risk device’ in the abstract were in three records not retrieved. These terms are indicative of generic adverse effects.

However, adverse effects specific to the individual paper were present in 32 of the 83 records not captured (Table A4). A search strategy which incorporates both generic and specific adverse effects terms could therefore potentially achieve 90% (447/498) recall in the validation set of records.

#### Summary

In summary therefore, the search filters (Box 1 and 2) retrieved 89%, 88%, 91% and 83% of the relevant records in medline and 89%, 88%, 85% and 83% of the relevant records in embase (Table 2). In each case, the addition of specific adverse effects terms could have improved the recall of the searches to 94%, 94%, 94% and 92% in medline and 95%, 93%, 93% and 90% in embase (Table 2).

## Discussion

We have used a cohort of included studies from systematic reviews on medical devices to derive and validate a novel search filter for the adverse effects of medical devices. The results here give an indication of performance in terms of relative recall of individual search terms and their combinations. The filters will also inevitably increase the precision of searches for adverse effects, although we were unable to quantify this.

We were able to compile a list of some of the specific terms commonly used in the databases and we recommend that searchers look to augment the search filter with these specific named adverse effects where appropriate. However, it is very apparent that the ‘specific’ terms are very narrow in scope and relevant only to a particular intervention, anatomical site and method of deploying the device. Unlike pharmaceutical preparations which typically are pill, potions, creams and injections, there is far greater diversity in how and where the device is fitted. Hence, the ‘specific’ AE are a mishmash that cannot easily be addressed by search filter terms. Therefore, reviewers could look at the physical characteristics and scientific development of the device, and pick out the most relevant specific adverse effects rather than rely on the specific terms listed in this paper. This would be best done by using our generic search filter and then adding those specific to site and device (e.g. cardiac tamponade for devices in the heart).

Search filters vary in the level of sensitivity and precision that can be achieved. Whilst we strive for 100%, generally lower levels of sensitivity are deemed acceptable and we adopted Benyon 2013's 90% or above threshold (Beynon et al., 2013). Perfect sensitivity is unachievable because some relevant records will always not contain any terms in the title, abstract or indexing to indicate they met certain criteria or present relevant data and examination of the full text will always be required. In addition, there is always a trade‐off between sensitivity and precision. The recall of searches using solely generic adverse effects terms was 84% in medline and 83% in embase. With the addition of specific adverse effects terms (to the generic adverse effects terms), the recall could be raised to 92% in medline and 90% in embase. The results for medical device searches here are less favourable compared with search filters for drug intervention adverse effects whereby sensitivity approaching 90% in both medline and embase was achieved without specific named adverse effects and 93% in medline and 96% in embase when specific adverse effects terms were added (Golder & Loke, 2012b). And also less favourable than searches for adverse effects of surgical interventions whereby sensitivity of 87% in medline and 92% in embase was achieved with generic adverse effects terms and 93% in medline and 95% in embase with the addition of specific adverse effects terms (Golder 2008). This is likely to be as a result of the more diverse adverse effects being associated with medical devices rather than for drug interventions and surgical procedures. Hence, there may be fewer generic terms useful for searching for general medical device adverse effects.

It should also be noted that the performance of the search filters for medical device adverse effects in the validation set in both medline and embase was poor in comparison with the test sets. However, when searching with only generic adverse effects terms, the sensitivity did not meet the 90% or higher target in the validation sets of records and five of the six test sets. The 90% target was however met for the test sets when generic and named adverse effects were searched in the validation set and all the test sets (Table 2).

We anticipate that these search filters will assist searchers when devising search strategies to identify relevant studies for a systematic review of the adverse effects of medical devices. In addition, we demonstrate the value of the addition of specific adverse effects terms where possible. However, we do not recommend these adverse effects filters for medical devices be used without due consideration, particularly as some of the search terms may only apply to certain types of medical device and that recent changes in indexing may impact on the performance. For instance, the recently introduced subheading in embase is ‘adverse medical effect (am)’ in March 2014.

Whilst the floating subheading adverse device effect (am.fs) is not currently included in our search filter, this is likely to be a result of the year of publication of many of our studies. This subheading was introduced in March 2014. Future research may see the value of this subheading for searching for adverse effects improve as it is more widely accepted and used.

## Limitations

A major limitation of the methodology used in this study is the lack of a true measurement of precision. We would need a large set of non‐relevant records in order to identify not just the most frequently occurring relevant terms but also the most discriminating terms and to measure precision. The current study simply indicates the relative rank precision of terms in relation to one another.

Our sample of records was obtained using search terms for both devices and safety in Epistemonikos. Although we included many synonyms and different devices, this may have limited the generalisability of our findings. The next steps in this area need to be the testing and validation on systematic review case studies (in which precision can be measured) and further research with larger sample sizes of relevant papers.

Medical devices have an added complexity in that they are often used in conjunction with another intervention. For instance, many medical devices require a surgical procedure for their placement such as breast implants and hip prosthesis. Other medical devices have a drug component embedded in them such as drug‐eluting stents. The diversity of types of medical devices and the common use of medical devices in conjunction with another type of intervention (such as pharmaceutical or surgical) meant that we employed a loose definition of ‘generic’ adverse effects terms. Some of the generic terms therefore are more specific to one type of device than another and may even be irrelevant to others.

## Conclusions

This is the first search filter for adverse effects of medical devices. The filter can be used where unmanageable numbers of records would otherwise be retrieved. Additional specific terms can be added to the filter to increase its sensitivity.

Further research on larger data sets is required in order to measure the precision of searching for adverse effects of medical devices and to test the suggested search filters with more rigour. In time with improvements in indexing and the adoption of subheadings such as ‘adverse device effects’ in embase, the sensitivity of future filters is likely to improve. Different categories of medical devices may require more individualised search filters.

## Funding

This paper presents independent research funded by a post‐doctoral fellowship PDF‐2014‐07‐041 through the National Institute for Health Research (NIHR). The views expressed are those of the author(s) and not necessarily those of the NHS, the NIHR or the Department of Health.

## Appendix

**Table A1:** Search terms in medline first test set of records (in order of relative recall)

Search term	Fields searched	Relative recall (*n* = 496)	Total number of records retrieved Medline 1946 to Present (18/04/2018)	Approximate relative precision estimate (%)	Relative recall * approximate relative precision (%)
Complicat*	Title/abstract	40% (196)	941 105	0.0208	0.00823
Complication*	Title/abstract	39% (194)	784 393	0.0247	0.00967
Adverse Effects (ae)	Subheading	36% (179)	1 569 747	0.0114	0.00412
Complications	Title/abstract	35% (174)	617 214	0.0282	0.00989
Exp postoperative complications/	MeSH	28% (139)	491 388	0.0283	0.00793
Safe*	Title/abstract	27% (135)	719 454	0.0188	0.00511
Failure	Subject heading word (HW)	23% (114)	333 438	0.0342	0.00786
Safety	Title/abstract	19% (95)	410 311	0.0232	0.00443
Failure*	Title/abstract	18% (91)	625 555	0.0145	0.00267
Complications	Subject heading word (HW)	18% (90)	549 931	0.0164	0.00297
Postoperative complications/	MeSH	17% (84)	330 594	0.0254	0.00430
Failure	Title/abstract	17% (83)	593 905	0.0140	0.00234
Exp equipment failure/	MeSH	15% (76)	82 988	0.0916	0.01403
Complication	Title/abstract	15% (73)	244 260	0.0299	0.00440
Adverse	Title/abstract	13% (63)	412 382	0.0153	0.00194
Safe	Title/abstract	11% (54)	304 054	0.0178	0.00193
Complication (co)	Subheading	10% (49)	1 826 599	0.0027	0.00027
Removal	Title/abstract	10% (48)	302 380	0.0159	0.00154
Adverse adj3 event*	Title/abstract	9% (47)	141 900	0.0331	0.00314
Failed	Title/abstract	7% (33)	252 342	0.0131	0.00087
Adverse event*	Title/abstract	5% (24)	124 887	0.0192	0.00093
Adverse events	Title/abstract	5% (23)	112 592	0.0204	0.00095
device removal/	MeSH	4% (22)	11 184	0.1967	0.00873
Loosen*	Title/abstract	4% (20)	18 225	0.1097	0.00442
equipment failure/	MeSH	4% (20)	22 205	0.0901	0.00363
loosening	Title/abstract	4% (19)	15 983	0.1189	0.00455
migration	Title/abstract	4% (19)	21 0297	0.0090	0.00035
Failures	Title/abstract	4% (19)	46 225	0.0411	0.00157
Problem*	Title/abstract	3% (17)	929 607	0.0018	0.00006
Rupture*	Title/abstract	3% (13)	113 170	0.0115	0.00030
Safe*	Subject heading word (HW)	3% (13)	99 952	0.0130	0.00034
Equipment Failure Analysis/	MeSH	2% (12)	36 533	0.0328	0.00079
Safely	Title/abstract	2% (10)	57 343	0.0174	0.00035
Malfunction*	Title/Abstract	2% (9)	13 078	0.0688	0.00125
Adverse adj3 reaction*	Title/abstract	2% (8)	42 035	0.0190	0.00031
Complain*	Title/Abstract	2% (8)	112 444	0.0071	0.00011
discomfort	Title/abstract	2% (8)	39 107	0.0205	0.00033
equipment safety/	MeSH	2% (8)	9925	0.0806	0.00130
exp Intraoperative complications/	MeSH	2% (8)	47 955	0.0167	0.00027
Problems	Title/abstract	2% (8)	502 026	0.0016	0.00003
Problem	Title/abstract	2% (8)	443 908	0.0018	0.00003
Side effect*	Title/abstract	2% (8)	219 091	0.0037	0.00006
Adverse effect*	Title/abstract	1% (7)	133 694	0.0052	0.00007
Adverse adj3 effect*	Title/abstract	1% (7)	155 139	0.0045	0.00006
Debris	Title/abstract	1% (7)	17 553	0.0399	0.00056
Side effects	Title/abstract	1% (7)	195 451	0.0036	0.00005
Adverse effects	Title/abstract	1% (6)	111 118	0.0054	0.00007
Adverse reaction*	Title/abstract	1% (6)	28 285	0.0212	0.00026
Complaint*	Title/Abstract	1% (6)	76 939	0.0078	0.00009
Tolerated	Title/abstract	1% (6)	125 288	0.0048	0.00006
Intraoperative complications/	MeSH	1% (5)	29 694	0.0168	0.00017
Adverse reaction	Title/abstract	1% (4)	6282	0.0637	0.00051
Complicat*	Keyword Heading Word (KF)	1% (4)	92 771	0.0043	0.00003
Adverse event	Title/abstract	1% (3)	20 626	0.0145	0.00009
Complicat*	Keyword Heading (KW)	1% (3)	11 308	0.0265	0.00016
Complication*	Keyword Heading (KW)	1% (3)	10 847	0.0277	0.00017
Failing	Title/abstract	1% (3)	23 592	0.0127	0.00008
Procedure related	Title/abstract	1% (3)	7763	0.0386	0.00023
Related morbidity	Title/abstract	1% (3)	7158	0.0419	0.00025
Safety/	MeSH	1% (3)	37 621	0.0080	0.00005
Adverse	Keyword Heading Word (KF)	0.4% (2)	9679	0.0207	0.00008
Adverse reactions	Title/abstract	0.4% (2)	23 285	0.0086	0.00003
Breakag*	Title/Abstract	0.4% (2)	13 819	0.0145	0.00006
complained	Title/abstract	0.4% (2)	22 502	0.0089	0.00004
Complications	Keyword heading (KW)	0.4% (2)	6886	0.0290	0.00012
Complications	Keyword Heading Word (KF)	0.4% (2)	87 076	0.0023	0.00001
Device related events	Title/abstract				
Displacement	Title/abstract	0.4% (2)	79 581	0.0025	0.00001
Failure	Keyword Heading Word (KF)	0.4% (2)	33 636	0.0059	0.00002
Failure*	Keyword heading (KW)	0.4% (2)	1075	0.1860	0.00075
Irritation	Title/abstract	0.4% (2)	19 763	0.0101	0.00004
Medical device recalls/	MeSH	0.4% (2)	157	1.2739	0.00514
Recall	Title/abstract	0.4% (2)	47 014	0.0043	0.00002
Safer	Title/abstract	0.4% (2)	29 653	0.0067	0.00003
Adverse effect	Title/abstract	0.2% (1)	262 94	0.0038	0.00001
Harm	Title/abstract	0.2% (1)	37 346	0.0027	0.00001
Harmful	Title/abstract	0.2% (1)	47 404	0.0021	0.00000
Impairing	Title/abstract	0.2% (1)	12 601	0.0079	0.00002
misplacement	Title/abstract	0.2% (1)	1597	0.0626	0.00013
Patient safety/	MeSH	0.2% (1)	14 124	0.0071	0.00001
product surveillance postmarketing/	MeSH	0.2% (1)	6525	0.0153	0.00003
Rupture/	MeSH	0.2% (1)	241 20	0.0041	0.00001
Safe*	Keyword Heading Word (KF)	0.2% (1)	19 761	0.0051	0.00001
Safe*	Keyword heading (KW)	0.2% (1)	9803	0.0102	0.00002
Safest	Title/abstract	0.2% (1)	3876	0.0258	0.00005
safety‐based medical device withdrawals/	MeSH	0.2% (1)	43	2.3256	0.00469
Side effect	Title/abstract	0.2% (1)	31 055	0.0032	0.00001
Side effects	Keyword Heading Word (KF)	0.2% (1)	7297	0.0137	0.00003

**Table A2:** Specific adverse effects terms identified in medline records not retrieved by generic adverse effects searches

Sets of records	Specific adverse effects terms
First test set	Abstract: aspiration, blood loss, blood staining, bone loss, bronchospasm, deformity, dental trauma, device lead defect, dislocation, erythema, groin pain, hemodynamic responses, dysphagia, dysphonia, inappropriate therapy [due to device], hoarseness, inappropriate shocks, infection, insertion pain, laryngospasm, metal ions, operative mortality, persistent pain, postoperative airway morbidity, regurgitation revision, subsidence, traumatic,, sore throat, tricuspid valve thrombosis, and valve deterioration. MeSH terms: dental restoration failure/, dental restoration wear/and heart valve prosthesis implantation/mo [mortality].
Second test set	Title: ‘sore throat’. Abstract: abnormal uterine activity, airway morbidity due to the devices, anastomotic leak, arterial dissections, bleeding, blood loss, bone loss, cesarean, deep infection, dislocation, dysphagia, inappropriate ICD shocks, in‐stent restenosis, lesions, marginal bone level alteration, pericardial tamponade, post‐operative peri‐abutment pin tract wound infection, revision, ominous foetal heart rate, postprocedural neurological deteriorations, malapposition, postoperative airway symptoms, stent thromboses, sore throat, and subsidence temporary phrenic nerve palsy, vessel perforations, vessel ruptures, and urinary leak. MeSH: dental restoration failure/, hoarseness/, and pharyngitis/
Third test set	Abstract: amenorrheic, audible component‐related noise, bleeding, bone loss, bone resorption, breast milk output, ceramic implant fracture, dislocation, oedema, haemorrhage, implant mobility, infection, infections, inflammation, lactational amenorrhoea, lactational performance, neonatal morbidity, neurological deficit [related to the depth electrode], revision, surgical and psychological distress, and weight gain. Specific adverse effect. MeSH: alveolar bone loss/, bone resorption/, dental restoration failure/, haemorrhage/, and menstruation disturbances/
Validation set	Title: dysphagia. Abstract: bleeding, blood loss, bone‐level change, cough, cystic lesions, death, dysphagia, inappropriate shocks, revision [e.g. lead dysfunction‐related revision], ICD storm, haemodynamic stimulation, minor trauma, bone loss, insertion pain, hoarseness, implant fracture, implant was lost, implantation morbidity, implants were lost, increased duration of pain, laryngopharyngeal symptoms, late luminal loss, mortality, neurological deficit, postoperative patellar crepitus, restenosis, revascularisation of the target vessel, risk of injury, scaffold thrombosis, sore throat, stent thrombosis, subluxation, target vessel revascularisation and uterine perforation MeSH: alveolar bone loss/and dental restoration failure/. Keyword headings (KW): CBD complications and dysphagia

**Table A3:** Search terms in embase first test set of records (in order of relative recall)

Search term	Fields searched	Relative recall (*n* = 496)	Total number of records retrieved embase 1946 to Present (22/06/2018)	Approximate relative precision estimate (%)	Relative recall * approximate relative precision
Complication (co)	Subheading	44% (219)	1 751 509	0.012504	0.005521
Complicat*	Title/abstract	43% (214)	1 341 114	0.015957	0.006885
Complication*	Title/abstract	42% (208)	1 140 438	0.018239	0.007649
Complications	Title/abstract	36% (181)	900 680	0.020096	0.007333
Exp complication/	EMTREE	34% (170)	961 554	0.01768	0.00606
Safe*	Title/abstract	31% (155)	1 085 522	0.014279	0.004462
Failure*	Title/abstract	21% (102	912 633	0.011176	0.002298
Safety	Title/abstract	20% (101)	637 622	0.01584	0.003225
Exp postoperative complication/	EMTREE	20% (98)	612 780	0.015993	0.00316
Exp medical device complication/	EMTREE	18% (88)	103 670	0.084885	0.01506
Complicat*	Heading Word (HW)	18% (88)	598 655	0.0147	0.002608
Complication*	Heading Word (HW)	18% (88)	598 481	0.014704	0.002609
failure	Title/abstract	18% (87)	870 531	0.009994	0.001753
Adverse	Title/abstract	15% (72)	638 560	0.011275	0.001637
Safe*	Heading word (HW)	14% (70)	712 834	0.00982	0.001386
Complication	Title/abstract	14% (69)	363 199	0.018998	0.002643
Failure*	Heading word (HW)	13% (63)	875 984	0.007192	0.000914
Exp safety/	EMTREE	12% (61)	438 680	0.013905	0.00171
Safe	Title/abstract	12% (60)	458 364	0.01309	0.001583
Postoperative complication/	EMTREE	12% (59)	316 632	0.018634	0.002217
Adverse adj3 event*	Title/abstract	12% (58)	253 031	0.022922	0.00268
Removal*	Title/abstract	8% (42)	382 901	0.010969	0.000929
Removal	Title/abstract	8% (41)	381 060	0.010759	0.000889
Failed	Title/abstract	6% (32)	331 417	0.009656	0.000623
Exp adverse event/	EMTREE	6% (30)	531 316	0.005646	0.000341
Adverse drug reaction (ae)	Subheading	6% (30)	1 241 745	0.002416	0.000146
Adverse event*	Title/abstract	6% (28)	222 968	0.012558	0.000709
Complicat*	Author Keyword (KW)	5% (26)	98 350	0.026436	0.001386
Complication*	Author Keyword (KW)	5% (26)	96 857	0.026844	0.001407
Safety/	EMTREE	5% (25)	250 238	0.00999	0.000504
Adverse device effect (am)[Link]	Subheading	5% (24)	30 291	0.079231	0.003834
Exp device removal/	EMTREE	5% (24)	23 689	0.101313	0.004902
Exp adverse device effect/	EMTREE	5% (23)	34 031	0.067585	0.003134
Adverse events	Title/abstract	5% (23)	199 706	0.011517	0.000534
Exp device infection/	EMTREE	5% (23)	303 94	0.075673	0.003509
Failures	Title/abstract	5% (23)	62 997	0.03651	0.001693
Complications	Author Keyword (KW)	4% (22)	80 367	0.027374	0.001214
medical device complication/	EMTREE	4% (21)	11 633	0.180521	0.007643
migration	Title/abstract	4% (21)	269 927	0.00778	0.000329
Device removal/	EMTREE	4% (20)	16 519	0.121073	0.004882
Problem*	Title/abstract	4% (20)	1 160 339	0.001724	0.0000695
Patient safety/(or exp patient safety)	EMTREE	4% (19)	94 286	0.020151	0.000772
Loosen*	Title/abstract	4% (18)	21 655	0.083122	0.003017
Device safety/(or explode device safety)	EMTREE	3% (17)	10 507	0.161797	0.005545
Safely	Title/abstract	3% (17)	86 511	0.019651	0.000674
loosening	Title/abstract	3% (16)	18 853	0.084867	0.002738
Problems	Title/abstract	3% (15)	637 650	0.002352	0.0000711
Side effect (si)	Subheading	3% (15)	832 846	0.001801	0.0000545
Drug safety/	EMTREE	3% (14)	321 156	0.004359	0.000123
Failure*	Author Keyword (KW)	3% (14)	102 190	0.0137	0.000387
Equipment safety/	EMTREE	2% (12)	9181	0.130705	0.003162
Rupture*	Title/abstract	2% (9)	140 438	0.006409	0.000116
peroperative complication/	EMTREE	2% (9)	35 983	0.025012	0.000454
Exp side effect/	EMTREE	2% (9)	493 291	0.001824	0.0000331
Complain*	Title/Abstract	2% (9)	176 729	0.005093	0.0000924
discomfort	Title/abstract	2% (9)	58 260	0.015448	0.00028
Complaint*	Title/Abstract	2% (8)	118 836	0.006732	0.000109
Adverse effect*	Title/abstract	1% (7)	1 852 63	0.003778	0.0000533
Adverse adj3 effect*	Title/abstract	1% (7)	214 172	0.003268	0.0000461
recall	Title/abstract	1% (6)	61 620	0.009737	0.000118
Adverse effects	Title/abstract	1% (6)	154 022	0.003896	0.0000471
Device infection/(under adverse device effect and medical device complication)	EMTREE	1% (6)	6785	0.08843	0.00107
Adverse adj3 reaction*	Title/abstract	1% (6)	66 676	0.008999	0.000109
Adverse event	Title/abstract	1% (5)	37 566	0.01331	0.000134
Adverse reaction*	Title/abstract	1% (5)	46 923	0.010656	0.000107
Debris	Title/abstract	1% (5)	22 088	0.022637	0.000228
Side effect*	Title/abstract	1% (5)	322 286	0.001551	0.0000156
Adverse	Candidate term word (DQ)	1% (5)	27 014	0.018509	0.000187
Device failure/	EMTREE	0.8% (4)	3667	0.109081	0.00088
Postoperative complications/	EMTREE	0.8% (4)	56 843	0.007037	0.0000568
Malfunction*	Title/Abstract	0.8% (4)	18 783	0.021296	0.000172
Side effect/	EMTREE	0.8% (4)	254 104	0.001574	0.0000127
Side effects	Title/abstract	0.8% (4)	286 457	0.001396	0.0000113
Problem	Title/abstract	0.8% (4)	5450 23	0.000734	0.00000592
Complication	Author Keyword (KW)	0.8% (4)	16 612	0.024079	0.000194
Adverse effects/	EMTREE	0.6% (3)	25 146	0.01193	0.0000722
Breakag*	Title/Abstract	0.6% (3)	164 66	0.018219	0.00011
Complicat*	Candidate term word (DQ)	0.6% (3)	15 177	0.019767	0.00012
Complication	Candidate term word (DQ)	0.6% (3)	1549	0.193673	0.001171
Tolerated	Title/abstract	0.6% (3)	197 595	0.001518	0.00000918
Adverse reaction	Title/abstract	0.6% (3)	10 517	0.028525	0.000173
Procedure‐related	Title/abstract	0.6% (3)	13 297	0.022561	0.000136
Related morbidity	Title/abstract	0.6% (3)	10 268	0.029217	0.000177
Adverse	Author Keyword (KW)	0.6% (3)	30 007	0.009998	0.0000605
Adverse	Heading word (HW)	0.4% (2)	271 787	0.000736	0.00000297
Adverse reactions	Title/abstract	0.4% (2)	35 818	0.005584	0.0000225
Device removal	Title/abstract	0.4% (2)	1081	0.185014	0.000746
Adverse reaction to metal debris/	EMTREE	0.4% (2)	34	5.882353	0.023719
Device recall/	EMTREE	0.4% (2)	382	0.52356	0.002111
Device related events	Title/abstract	0.4% (2)	118	1.694915	0.006834
Safe*	Author Keyword (KW)	0.4% (2)	48 274	0.004143	0.000017
Irritation	Title/abstract	0.4% (2)	27 511	0.00727	0.000029
Failure*	Candidate term word (DQ)	0.4% (2)	8804	0.022717	0.000092
Safe*	Author Keyword (KW)	0.4% (2)	48 274	0.004143	0.000017
misplacement	Title/abstract	0.4% (2)	2189	0.091366	0.000368
Adverse effect	Title/abstract	0.2% (1)	36 458	0.002743	0.000006
Adverse outcome	Title/abstract	0.2% (1)	11 431	0.008748	0.000018
Adverse outcome/	EMTREE	0.2% (1)	39 136	0.002555	0.000005
absence of complications/(under complication/)	EMTREE	0.2% (1)	1176	0.085034	0.000171
Absence of side effects/	EMTREE	0.2% (1)	17 796	0.005619	0.000011
Equipment failure/	EMTREE	0.2% (1)	5076	0.019701	0.000040
Failing	Title/abstract	0.2% (1)	33 458	0.002989	0.000006
Harm	Title/abstract	0.2% (1)	49 694	0.002012	0.000004
Harms	Title/abstract	0.2% (1)	14 403	0.006943	0.000014
Impairing	Title/abstract	0.2% (1)	17 037	0.00587	0.000012
Malfunction*	Candidate term word (DQ)	0.2% (1)	386	0.259067	0.000522
Risk benefit analysis/(under risk)	EMTREE	0.2% (1)	50 973	0.001962	0.000004
Safer	Title/abstract	0.2% (1)	41 343	0.002419	0.000005
Safest	Title/abstract	0.2% (1)	5514	0.018136	0.000037
Side effect	Title/abstract	0.2% (1)	47 920	0.002087	0.000004
Side effects	Author Keyword (KW)	0.2% (1)	8055	0.012415	0.000025
complained	Title/abstract	0.2% (1)	35 959	0.002781	0.000006
Complication/	EMTREE	0%	124 785	0	0%
Adverse event/	EMTREE	0%	14 409	0	0%
Adverse device effect/	EMTREE	0%	4733	0	0%
Equipment safety	Author Keyword (KW)	0%	77	0	0%
adverse drug reaction/	EMTREE	0%	212 066	0	0%

^†^As the subheading ‘adverse device effect’ was introduced in March 2014 we calculated the sensitivity of this term limited to 2014, 2015, 2016, and 2017 in the Date Created field. Overall a search limited to date created 2014 onwards retrieved – 24/126 (19%) records. 8/55 (15%) Records created in 2014 were retrieved with this term. 13/55 (24%) in 2015, 2/15 (13%) in 2016, 1/2 (50%) in 2017. This would have made the subheading ‘adverse device effect’ 10^th^ in terms of highest sensitivity terms.

**Table A4:** Specific adverse effects terms identified in embase records not retrieved by generic adverse effects searches

Sets of records	Specific adverse effects terms
First test set	Abstract: blood loss, bone loss, calcified, cardiac death, cystic lesions, component malposition, degeneration, drill holes, dysphagia, erythema, inappropriate ICD discharges, infection, loss of lordosis, luminal loss, major thromboembolism, mortality, restenosis, revascularisation of the target lesion, revision, sore throat, target‐lesion revascularisation, subsidence, traumatic, unilateral capsular contractions, and valve‐related re‐operation. Emtree: dysphagia/, degradation/, infection/, restenosis/, sore throat/, and wound healing/.
Second test set	Title: skin breakdown. Abstract; bleeding, blood on the device, bone level change, bone loss, cage subsidence, defective lead, device/lead defect, dysphagia, high metal ion levels, inappropriate shocks, implantation morbidity, inappropriate therapy, inappropriate treatment shock, in‐stent late loss, insulation breach, insulation defects, luminal loss, minor trauma, neurological deficit, postoperative low cardiac output, pseudotumours, restenosis, revision, skin compromise, target vessel revascularisation, silicone synovitis, thrombosis tunnel ossification, tunnel widening and uterine perforation. Emtree: skin irritation/, trace metal blood level/, and uterus perforation/.
Third test set	Abstract: aberrations, aseptic meningitis, bone loss, bone resorption, central‐line associated bloodstream infection, contrast sensitivity cognitive effort, cobalt levels, compromising adj4 swallowing function, compromising voice quality, cough, deep wound infections, died, displacement, dysphagia headache, hoarseness, IABP‐related morbidity ICD storm, implant* adj2 lost, inappropriate shocks, in‐hospital morbidity, late luminal loss, laryngopharyngeal symptoms, lead dysfunction‐related revision, loss adj2 cartilage, loss adj2 mucosa, loss adj2 tissue, more need for oxytocin augmentation,, mortality, occlusion, pain response, pain scores, pain with insertion, periprosthetic severe regurgitation, persistent pain, physically taxing, Postoperative cerebrospinal‐fluid (CSF) leaks, postoperative intensive care unit stay, radiolucency, re‐bleeding, recurrent caries, recurrent stenosis, serum chrome, serum ion levels, sore throat, stress responses, subcutaneous cerebrospinal‐fluid accumulation, thrombosis, transfusion, tissue loss, vena caval penetration, venous pressure, wound infection). Emtree: alveolar bone loss/and crestal bone loss/.
Validation set	Title: ‘degradation’. Abstract: arrhythmogenic morbidity, blood loss, bone loss, dysphonia, cable extrusion, caesarean, capsular contracture, cerebral abscesses, contamination, cough, crestal bone loss, cyst formation,, degradation, degraded, edge dissection foreign body reactions, haemorrhage, haemodynamic changes, haemodynamic profiles, incomplete strut apposition, increased duration of pain, infection, insertional pain, ischaemia, ischaemic events, Leakage, loss of integration, inappropriate shocks, marginal bone loss neurological deficit, phrenic nerve palsy, pericardial tamponade,, residual area stenosis, rhinorrhea, risk of injury, screw breakages, sore throat, strut fracture, subdural hematoma, subsidence, tissue prolapse, tunnel widening, uterine tachysystole, uterine perforation, and urine leak. Emtree: alveolar bone loss/, Contraception‐‐side effects/, Contraceptive Methods‐‐side effects/, degradation/, heart tamponade/, phrenic nerve paralysis/, prosthesis failure/, target vessel failure/and target lesion revascularisation/.
